# Survival Prognostic Factors of Non-Invasive Ventilation in Amyotrophic Lateral Sclerosis: A Systematic Review

**DOI:** 10.3390/life14121664

**Published:** 2024-12-16

**Authors:** Aleksandra Orlova, Yaroslav Malygin, Anna Gofman, Sofija Sotulenko, Veronika Gandalian, Ioan Kartashov, Lev Brylev, Sergey Bolevich, Tamara Nikolic Turnic, Vladimir Jakovljevic

**Affiliations:** 1 Department of Pathological Physiology, Institute of Digital Biodesign and Modelling of Living Systems, I.M. Sechenov First Moscow State Medical University (Sechenov University), 119991 Moscow, Russia; orlova_a_s@staff.sechenov.ru (A.O.); kartashov_i@yandex.ru (I.K.); bolevich2011@yandex.ru (S.B.); drvladakgbg@yahoo.com (V.J.); 2 Faculty of Fundamental Medicine, Lomonosov Moscow State University, 119991 Moscow, Russia; malyginyv@my.msu.ru; 3 Institute of World Medicine, Pirogov Russian National Research Medical University, 117997 Moscow, Russia; makabi2806@gmail.com (A.G.); sotulenko_sofiya@mail.ru (S.S.); nika.gandalian@gmail.com (V.G.); 4 Yas Clinic Managed by Abu Dhabi Stem Cell Center, Abu Dhabi, United Arab Emirates; lev.brylev@adscc.ae; 5 Moscow Research and Clinical Center for Neuropsychiatry, Moscow Healthcare Department, 127006 Moscow, Russia; 6 Department of Pharmacy, Faculty of Medical Sciences, University of Kragujevac, Svetozara Markovica 69, 34000 Kragujevac, Serbia; 7 N.A. Semashko Public Health and Healthcare Department, F.F. Erismann Institute of Public Health, I.M. Sechenov First Moscow State Medical University (Sechenov University), 119435 Moscow, Russia; 8 Department of Physiology, Faculty of Medical Sciences, University of Kragujevac, Svetozara Markovica 69, 34000 Kragujevac, Serbia

**Keywords:** systematic review, amyotrophic lateral sclerosis, prognostic factors, survival, non-invasive ventilation

## Abstract

Objective: Amyotrophic lateral sclerosis is a neurodegenerative disease with high rates of disability and mortality. Non-invasive ventilation (NIV) is an effective method of treating patients, increasing life expectancy, but currently, predictors available to determine the best outcome of therapy in this category of patients are unknown. This systematic review aimed to determine the impact of prognostic factors on benefits from NIV application compared with non-NIV tools of treatment (invasive ventilation and standard care) in case of survival of ALS patients. Method: We systematically sought relevant longitudinal cohort and case-control studies published in PubMed, CINAHL/EMBASE, Cochrane library, and Scopus. Results: We included seven prospective studies, published in 2010–2020, in the analysis. According to the evidence base available to date, NIV favors survival compared to non-NIV in patients with bulbar onset ALS. We obtained conflicting data on the significance of spinal onset and bulbar function. Survival depending on patient age, and also for spinal, cervical, and flail limb phenotypes during NIV therapy has not been sufficiently studied and needs further investigation. Conclusions: The studies analyzed in this review allow us to state with confidence that NIV is effective in bulbar onset ALS, taking into account recommendations for duration of ventilation and the use of the full range of symptomatic therapy, including mechanically assisted coughing. The effectiveness of NIV on severe bulbar symptoms requires further research.

## 1. Introduction

Amyotrophic lateral sclerosis (ALS) is a neurodegenerative disease characterized by progressive loss of upper and lower motor neurons, resulting in weakness of somatic muscle tissue and impairment of vital functions [1]. Epidemiological studies show that the incidence of ALS in the world varies from 0.6 to 3.8 cases per 100,000 population. The highest incidence is registered in Sweden (Stockholm) and Scotland with 3.8 cases per 100 thousand population; the lowest is registered in South Korea and China (1.2 and 0.8 cases per 100 thousand population, respectively) [2].

All clinical types of ALS ultimately result in severe bulbar impairment. Bulbar dysfunction in ALS affects respiration by reducing caloric intake and causing weight loss, increasing the risk of infection, compromising airway protection, leading to aspiration, and contributing to fibrosis and increased risk of death [3]. Bulbar dysfunction also affects the tolerability of non-invasive ventilation and influences ventilation parameters [4]. The decline in respiratory muscle function leads to increased work of breathing, microatelectasis from retention of mucus, and subsequent decrease in lung compliance [5]. Indeed, the most frequent cause of death in ALS patients is respiratory insufficiency secondary to respiratory musculature impairment [6]. Treatment is difficult as no method exists to regain muscle function. Disease progression ultimately leads to ventilation assistance. Although this has traditionally been achieved with invasive ventilation via tracheostomy, several studies highlight its shortcomings. Tracheostomy has been associated with long-term morbidity as well as increased burden of care [7]. Non-invasive ventilation (NIV) has emerged as a promising alternative with studies showing increasing use in multiple countries [8].

Currently, there are recommendations that converge on the main parameters of respiratory parameters that indicate the use of non-invasive ventilation in ALS. However, in actual clinical practice, the use of ventilation depends on many factors: the availability of equipment, specialists, organizational opportunities, and cultural characteristics [9,10]. Additionally, the use of NIV significantly alters the patient’s usual way of life and in most cases requires the involvement of other family members [11]. The decision-making process for initiating NIV in ALS is complex and evolves over time, often shaped by the unpredictable and progressive nature of ALS [12,13,14]. Studies examining patients’ decisions to initiate non-invasive ventilation (NIV) have highlighted the need to consider patients’ views on prolonging life, their fear of the procedure and their understanding of NIV [15]. Therefore, data on the impact of NIV on survival in individual patients are a critical part of decision making.

We conducted this review with the aim to determine the impact of prognostic factors on benefits of NIV application compared with non-NIV tools of treatment (invasive ventilation and standard care) in case of survival of ALS patients.

## 2. Materials and Methods

### 2.1. Protocol and Registration

The present systematic review was conducted following the PRISMA 2020 statement and guidelines for systematic reviews and meta-analyses of prognostic factors [16,17,18]. The protocol used for this systematic review was registered on PROSPERO (PROSPERO 2022 CRD42022354654) [19]. This systematic review identified and appraised observational studies published between examining factors related to survival of patients with ALS on NIV and factors improving it.

### 2.2. Eligibility Criteria

Inclusion criteria for this review necessitated that the publication should contain the following:

(1) Availability of full text;
(2) English language, Russian language;
(3) Empirical data;
(4) Peer-reviewed journal;
(5) Cross-sectional study, retrospective study, cohort longitude study, randomized control trials, postal surveys;
(6) Analysis of survival in patients at NIV vs. non-NIV is performed;
(7) While conducting survival analysis patients must be divided at least at 2 subgroups according to their characteristics (demographic, clinical etc.).

Authors excluded studies that employed non-human participants. Systematic reviews, meta-analyses, case-reports, studies without abstracts, notes, or letters were also excluded.

### 2.3. Information Sources

The literature search was conducted from 1 to 10 September 2024 within the usage of the following databases: PubMed, CINAHL/EMBASE, Cochrane library, and Scopus. There were no limits on the date of issue of the publications. Besides, hand searching was performed using articles from the journal *Amyotrophic Lateral Sclerosis and Frontotemporal Degeneration* from the period 2000–2024 in order to identify other eligible studies.

### 2.4. Search Strategy

The search strategy and terms were developed by three authors (Y.M., A.O. and L.B.). The authors are trained in the search for literature, two of them are neurologists (A.O. and L.B.), and one of them (L.B.) is a specialist in the field of AML.

Two independent authors (A.G., S.S.) searched PubMed, Cochrane library, CINAHL/Embase, and e-Library databases for relevant articles on the subject of ALS, non-invasive ventilation, and survival until 7 March 2021. Searchers also involved combinations of keywords and Medical Search Headings (MeSH terms) such as the following:

Amyotrophic Lateral Sclerosis [*mortality]; Amyotrophic Lateral Sclerosis/therapy*, Motor Neuron Disease [mortality]; Motor Neuron Disease/complications*, Respiration, Artificial/adverse effects, Respiratory Therapy/methods*, Noninvasive Ventilation/methods*, Noninvasive Ventilation/trends*, Randomized Controlled Trials as Topic; Respiration, Artificial [methods] [*mortality]; Respiratory Insufficiency [etiology] [*mortality] [therapy]; Dyspnea/[epidemiology] [therapy], Positive-Pressure Respiration/methods*, Positive-Pressure Respiration/statistics and numerical data*, Survival Analysis; Time Factors.

### 2.5. Selection Process

Authors of this review included the following types of research: cross-sectional studies, retrospective studies, cohort longitude studies, randomized control trials, postal surveys, longitudinal studies, and case studies.

After searching databases, three authors (A.G., S.S., and V.G.) conducted a critical reading of the titles and review of the abstracts to identify relevant articles according to the inclusion criteria. They independently reviewed and then sought agreement. In cases of unanimous opinions, articles were classified as included or excluded. In cases of discordance between authors, articles were classified as doubt. Those that generated doubts were evaluated by three co-investigators who were not participating in the primary reading (A.O., Y.M., and L.B.) to determine, by consensus, a final decision.

The full text of the publications were assessed by three separate researchers (A.G., S.S., and V.G.) using the inclusion/exclusion criteria. Three researchers independently reviewed the full text of the publications and evaluated the type of study, subjects, the differences between NIV and non-NIV treatment strategies in ALS patients’ outcomes, and interpretated the results. Based on the search results, researchers compiled a database in which they indicated publication data, population included, estimation methods, and results. When the reported data were unclear, a third co-investigator (Y.M.) participated in reviewing and evaluating the data for inclusion.

### 2.6. Data Collection Process

Three review authors (A.G., S.S., and V.G.) independently extracted data from the full text of included studies to the Excel spreadsheet with the following information: first author, review search dates, study design, number of patients undergoing NIV and non-NIV therapeutic modes, outcome of interest (survival under each method), and list of possible factors improving NIV with the corresponding survival. Collected data were reviewed and discussed by the two co-investigators (A.O. and Y.M.). All of the data were discussed by the research team and agreed upon.

### 2.7. Data Items

Data from included studies were sought for the following outcomes: survival rates of ALS patients on NIV compared with those on non-NIV, and also the survival rates of ALS patients depending on factors influencing application of NIV.

From each included study the following data were extracted:

(1) Bibliometric details of the publication (first author name, year of publication);
(2) Study characteristics (country, study design, sample size, follow-up period, N at follow-up, and %);
(3) Participant characteristics (mean age and range, male/female ratio);
(4) Effect of predictor at outcome (direction of effect and *p*-value).

### 2.8. Synthesis Methods

Results were therefore presented in two analyses. Firstly, we described comparison between survival rates of ALS patients on NIV and non-NIV treatment.

Also, in this review, different factors which can have influence on NIV treatment were assessed. We performed survival rates on application of NIV in ALS patients depending on factors that influenced NIV treatment (e.g., age, body mass index, FVC values etc.). The data obtained were then summarized in narrative form.

### 2.9. Dealing with Missing Data

We contacted investigators in order to verify key study characteristics and to obtain missing numerical outcome data. During the data extraction, researchers obtained the missing numerical outcome data concerning survival on the NIV or non-NIV management, or accurate numerical survival on the factors improving NIV. As the contact with investigators was impossible to be achieved, the missing data were marked as “unknown” or “not established”.

### 2.10. Study Risk of Bias Assessment

Two review authors (S.S. and V.G.) then assessed the risk of bias in the studies using a standardized tool for randomized control studies—Version 2 of the Cochrane risk of bias tool for randomized trials (RoB 2) and non-randomized studies—Cochrane the Risk of Bias assessment tool in non-randomized studies of interventions (ROBINS-I tool) [20]. The quality of risk of bias assessment was performed in a consensus authors meeting and with the involvement of the one arbitrator (A.O.).

Any disagreements about inclusion of the articles in the systematic review were resolved by discussion between review authors. If a consensus was not achieved, the disagreements were resolved through the involvement of one arbitrator (A.O.).

We evaluated random sequence generation, allocation concealment, blinding (participants, personnel, and outcome assessors), incomplete outcome data, selective outcome reporting, and other sources of bias. We then made a judgement on each of these criteria of ‘high risk’, ‘low risk’, or ‘unclear risk’ that indicates an unclear or unknown risk of bias.

Details on algorithm of bias evaluation and results on evaluation of each article can be provided by the corresponding author by an e-mail request.

## 3. Results

### 3.1. Search Results

Briefly, the literature search identified 1131 articles (1088 from the database searching, 43 were accepted from the other source—journal “*Amyotrophic Lateral Sclerosis and Frontotemporal Degeneration*”). The following citations underwent screening during which 755 duplicates were removed. The inclusion and exclusion criteria were applied and used to screen 397 abstracts, of which 254 articles were selected for full-text screening. Finally, seven articles were included in this review.

**Figure 1:**
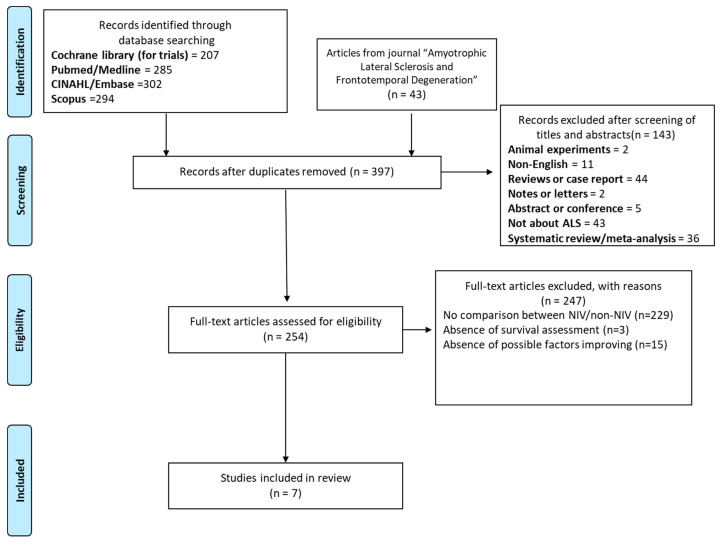
Flow diagram of identification and selection of included studies.

The study search, selection process, and reasons for excluding studies are summarized in Figure 1.

### 3.2. Identification of the Relevant Studies

Our initial database search retrieved 1131 eligible studies (1088 from the database searching, 43 were accepted from the other source—journal “*Amyotrophic Lateral Sclerosis and Frontotemporal Degeneration*”). Exclusion of 755 duplications, 397 irrelevant studies based on the title and abstract, and 254 studies after full-text reading left seven suitable studies.

### 3.3. Characteristics of the Included Studies

Of the seven studies, four were from Europe, two were from the USA, and one was from Australia. Most studies were published between 2010 and 2020. One study was prospective, another five were retrospective, and one randomized controlled trial was also included in this systematic review. Sample size of the studies varied from 18 to 919 patients. Duration of observation depended on number of patients included in the study and varied from 8 to 240 months.

An overview of the included studies is presented in Table 1.

### 3.4. Quality of the Included Studies

The assessment of the risk of bias of the included studies used a standardized tool for randomized control studies—RoB 2—and for non-randomized studies, ROBINS-I tool was used; shown in Table 2.

The studies were judged to have a risk of bias in study selection (random sequence generation and allocation concealment), attrition, statistical analysis, and reporting.

All studies, except one by Bourke [13], were classified as having a high risk of bias for study selection because they did not use randomization. All of the studies were evaluated as low risk for the attrition bias, as there were no missing outcome measures that can bias the results. In addition, each of the included studies was considered to be at low risk of reporting bias, because all the data that were claimed to be analyzed were presented. The diagnostic and survival analysis methods used in all articles were characterized as having high validity, resulting in low bias in outcome measurement. But nevertheless, most of the studies with the exception of ones conducted by Sancho and Siirala were evaluated as high risk for other bias because in all of them patients with slow disease progression were not excluded from the analysis.

### 3.5. Influence of NIV on ALS Patients’ Survival

All of the included studies except that conducted by Ackrivo J et al. [26] showed significantly increased survival on NIV treatment compared to ALS patients who had not undergone NIV or received conservative therapy. The results are showed in Table 3.

### 3.6. Patient Factors That Influence Survival on NIV Optimization

#### 3.6.1. Age

One of the included studies analyzed the effect of age on tolerance to NIV treatment. The study conducted by Siirala et al. has investigated the effect of NIV treatment in younger and elderly groups, separately, and has found out that NIV treatment was significantly effective in the older group (65 and older) compared to conservative treatment [21].

#### 3.6.2. Disease Onset

Five of the included studies have investigated the influence of the disease onset on benefits from the NIV treatment. Four studies showed that patients with bulbar onset had longer survival with NIV treatment compared with non-NIV treatment [21,22,23,25]. In contrast, the study by Ackrivo J. et al. found no significant difference between bulbar onset and survival on NIV, but this study was characterized by multiple high risks of bias [26]. Also, four of the included studies investigated the implementation on the patients with limb onset [21,23,25,26]. Additionally, Khamankar N. et al., Ackrivo J. et al., and Jesus S. et al., found out that those with spinal onset showed a significant increase in survival rates with NIV [23,24,26], however, Walsh LJ et al. did not find significant improvement of survival among the patients with spinal onset during NIV [25].

In their study, Berlowitz DJ et al. stated that cervical and flail limb phenotypes were not associated with longer survival with NIV treatment compared to non-NIV therapy [22].

#### 3.6.3. Bulbar Function

More specifically, the effect of bulbar dysfunction on NIV tolerance was investigated in two studies included in this systematic review. Jesus S et al. showed that patients with moderate and severe bulbar dysfunction survived significantly longer on NIV compared with the non-NIV group [24]. However, Bourke SC et al. found out that patients with severe bulbar dysfunction had no significant differences in survival duration on NIV, but showed some benefit in terms of quality-of-life measures, including µsym (*p* = 0.018) [13]. However, it is important to note that the study by Bourke SC et al. is considered a low risk study when compared to Jesus S et al. in the risk of bias assessment [13,24].

#### 3.6.4. Quality of Life

A study by Bourke SC et al. showed that NIV improved quality of life in patients with ALS [13]. An improvement in the quality of life was observed in patients with good bulbar function, while the quality of life in patients with poor bulbar function on NIV was comparable to standard treatment. However, of the studies we analyzed, this was the only one where the authors paid attention to this aspect of the patients’ condition.

A summary of all patient factors that can impact NIV tolerance and influence its optimization can be found in Table 1.

## 4. Discussion

This review highlights the importance of NIV in survival among the patients with ALS. The main challenges of using non-invasive ventilation (NIV) in ALS include the variable availability of equipment and qualified personnel, the long-term impact of ventilation on quality of life, the progressive nature of the disease requiring constant adjustment of ventilation parameters, and the gradual increase in the duration of ventilation. All this requires a thorough analysis of the available information on all aspects of NIV in ALS in order to provide it to the patient as part of the shared decision-making process [14]. It will become even more complicated as we obtain more data on the effectiveness of NIV in patients without symptoms of impaired breathing function in the early stages of the disease [27].

Almost all of the studies included in our systematic review found out that the survival level among ALS patients who received NIV was higher compared to those who had undergone conservative treatment, and it was statistically valid which was demonstrated in seven out of eight studies included in this current review.

Our systematic review of controlled studies allows us to shed more light on the effectiveness of ventilation in different patient groups, primarily in patients with bulbar impairments.

It is important to note that some of the studies we analyzed assessed the effect of disease onset, while others assessed the severity of bulbar symptoms. Among patients with the bulbar onset of the disease, three out of four studies demonstrated the effectiveness of ventilation in terms of life extension, with only one study not finding a positive effect of ventilation in patients with a bulbar onset. Of the two studies evaluating the effectiveness of ventilation in severe bulbar symptoms, one study did not achieve effectiveness; it was the randomized study by Bourke SC (2006), which is referenced in the European guidelines [12].

In our opinion, there are two main reasons for the negative results of ventilation both in patients with bulbar onset and those with severe bulbar impairments. The first reason is the inclusion of patients in studies who used ventilation for less than 4 h a day; both studies with negative results had a significant proportion of patients with ventilation for less than 4 h (Table 1). Several studies have shown significantly less effect of ventilation with such low usage time [24,28,29]. In the studies by Walsh LJ and Jesus S, ventilation for less than 4 h a day was an exclusion criterion [24,25], while in the Siirala W study, patients with ventilation for less than 4 h were included in the control group [21]. Bulbar symptoms reduce compliance with NIV [28], and low ventilation duration is one of the signs of poor tolerance of ventilation [30]. Excluding patients with low ventilation duration from studies can demonstrate its positive effect.

The second factor that we believe makes ventilation effective in bulbar impairment is the use of mechanically assisted coughing (MAC). MAC was used in the study by Jesus S, in 76.7% of patients who had severe bulbar impairments [24], while in the Bourke study, patients used MAC only at the end of the study, when it was licensed in UK [13]. According to existing recommendations, an airway clearance device is indicated for patients with weakened cough as a therapy that helps clear mucus and reduce the risk of pneumonia [24,31]. The use of MAC in conjunction with NIV can have a positive effect on the lifespan of patients with neuromuscular diseases [32].

It is worth mentioning separately the size of the populations studied in the Bourke study. The Bourke study analyzed 41 patients and the authors themselves acknowledge the low statistical power of the study to detect the ineffectiveness of NIV in severe bulbar symptoms [13].

Therefore, at present, the studies analyzed in this review allow us to state with confidence that NIV is effective in ALS with bulbar onset, provided that recommendations for duration of ventilation and use of the full range of symptomatic therapy, including MAC, are followed. The effectiveness of NIV in patients with severe bulbar symptoms requires further research.

To the best of our knowledge, this is the first systematic review of the impact of different factors on survival in ALS patients with NIV, as other systematic reviews have analyzed the benefit of using this treatment regardless of patients’ clinical characteristics as well as technical features of the technique to improve therapeutic outcomes [33,34].

However, multiple limitations collide the efforts to establish an adequate protocol for NIV management. The first problem is in the nature of ALS. Heterogeneity of disease presentation (e.g., differences in values of such factors as age, disease onset, and grade of bulbar function between investigated groups in all included studies) as well as variable rates of progression make it difficult to establish a guideline that uniformly applies to all cases.

Another limitation is in the lack of research performed on non-invasive ventilation among ALS patients and the implication of the factors that can influence the results of NIV use. Small sample size has been pointed out as a significant limiting factor in several studies.

As for recommendation for future studies, it is necessary to investigate the influence of the included factors (age, bulbar function, limb onset, spinal phenotype, cervical, and flail limb phenotypes) on survival and correlation with nutritional aspects, timing of installation of non-invasive ventilation in relation to respiratory assessment measures (for example, Forced Vital Capacity).

The comparative effect of NIV and non-NIV needs to be studied not only on survival rate during NIV treatment but also for the quality of life of ALS patients. It would also be interesting to evaluate the impact of NIV itself on the quality of life of ALS patients.

### Recommendations for Clinicians

The recommendations by A. Khan et al. (2023) cover all neuromuscular diseases for which non-invasive ventilation is indicated [35]. Our systematic review examines the existing publications on factors that influence the life expectancy of patients with amyotrophic lateral sclerosis who are ventilated. The main practical conclusion of our review is consistent with the recommendations of the referenced source [35], which also emphasizes that ventilation should be offered when indicated, regardless of bulbar impairment. However, our review provides additional material for practitioners to build a dialogue with the patient and their family during the process of making joint decisions about initiating ventilation in an individual clinical situation.

When discussing the decision to start NIV with the ALS patient and his family, it should be borne in mind that NIV in bulbar form prolongs the patient’s life.

## 5. Conclusions

ALS is a progressive neurodegenerative illness that eventually leads to respiratory impairment. NIV treatment is strongly associated with longer survival in ALS patients compared with conservative non-NIV treatment.

In this systematic review it was found that NIV use has been shown to have benefits on survival in patients with bulbar onset. Benefits of NIV in patients with severe bulbar symptoms are controversial.

## Figures and Tables

**Table 1:** Characteristics of studies of ALS patients’ survival on NIV treatment included in the systematic review.

Authors	Country	Study Design	Duration of Observation	Sample Size and Characteristics	Patients’ Characteristics	Intervention Description/Inclusion-Exclusion Criteria	Factor Tested for Effect at Survival
Bourke SC et al., 2006 [13]	United Kingdom	Randomized controlled trial	1 year/12 months	Sample size: 41 patients NIV 22 Non-NIV 19	NIV Male—14 Female—8 Age—63.7 ± 10.3 years Non-NIV Male—10 Female—9 Age—63.0 ± 8.1 years	NIV was initiated in hospital by use of a pressure-support ventilator in spontaneous/timed mode. Inspiratory and expiratory airway pressures were adjusted for optimum nocturnal oximetry breathing room air, daytime arterial blood gases. In those with poor upper limb function but relatively preserved bulbar function, the use of a mouthpiece positioned where the patient could reach it with his or her mouth, with the ventilator set to “smart on/off”, proved a useful means of conferring independence and optimizing compliance. Exclusion criteria: usage of NIV before randomization, absence of symptomatic daytime hypercapnia, Pmax more than 60%.	Bulbar function
Waltteri Siirala et al., 2013 [21]	Finland	Registry-based retrospective cohort study, non-randomized	11 years/135 months	Sample size: 84 NIV—41 Non-NIV—43 Both groups were subdivided based on patients’ ability to tolerate the NIV.	NIV Male—19 Female—22 Non-NIV Male—18 Female—25	NIV was given using a pressure-assisted ventilator. The average weekly duration of NIV use was collected using the device’s in-built counter, normally at 3-month intervals. Patients undergoing NIV less than 4 h per day at the last control visit, timed 1 week to 3 months prior to death, were considered NIV-intolerant and were allocated to the Conventional Group.	Age
Berlowitz DJ et al., 2015 [22]	Australia	Randomized controlled trial (RCT)	20 years/240 months	Sample size: 919 NIV—208 Non-NIV—711 The NIV group was formed of those patients who tolerated NIV treatment at least until first follow-up appointment. The non-NIV group was formed of those patients who did not.	NIV Male—157 Female—62 Age—58.3 ± 11.5 years Non-NIV Male—375 Female—335 Age—64.0 ± 11.9 years	Criteria for offering NIV initiation were orthopnea or sniff nasal pressure <40 cm H_2_O or maximal inspiratory pressure (MIP) <−60 cm H_2_O or abnormal nocturnal oximetry or vital capacity <50%). Patients were treated with NIV proceeded by bi-level pressure ventilator in spontaneous timed mode with a face mask. If a patient cannot be adequately ventilated non-invasively, ventilation via a tracheostomy was discussed or conservative palliative treatment was only prescribed.	Type of onset
Khamankar N. et al., 2018 [23]	USA	Retrospective cohort study	16 years/192 months	Sample size: 460 NIV—391 Non-NIV—69	No group characteristics	“Non-users” never used Bi-PAP at any point during their disease duration and “users” consistently used Bi-PAP on a daily basis for > 3 months prior to death.	Type of onset
Jesus Sancho et al., 2018 [24]	Spain	Prospective non-randomized	3 years/36 months	Sample size: 140 120 NIV 20 non-NIV The NIV group was formed of those patients who agreed to NIV treatment. The no-NIV group was formed of those patients who refused treatment with NIV.	NIV Male—56 Female—64 Age—64.05 ± 9.11 years Non-NIV Male—6 Female—14 Age—66.05 ± 10.27 years	In those subjects in whom symptoms of hypoventilation, hypercapnia, or respiratory accessory muscle use persisted during the daytime despite effective nocturnal NIV, daytime NIV was provided via a mouthpiece (Philips Respironics), lip seal mouthpiece or nasal pillow interface, or Air Liquide Healthcare as appropriate. Continuous NIV was defined as taking place when the ventilator was used for >20 h per day. Exclusion criteria was the use of NIV for <4 consecutive hours at night.	Bulbar dysfunction
Walsh LJ et al., 2021 [25]	Ireland	Retrospective chart analysis	6 years/72 months	Sample size: 111 patients NIV 74 Non-NIV 37	Age 63.83 ± 11.4 years NIV Male—57 Female—17 Non-NIV Male—11 Female—26	Optimal use of NIV was defined as use of the machine for greater than 4 h per day and for greater than 70% of the days used. Exclusion criteria: usage of NIV less than 4 h per day and less than 70% of the days used.	Type of onset
Ackrivo J et al., 2021 [26]	USA	Single-center retrospective cohort study	10 years	Sample size: 452 NIV—272 Non-NIV—180	NIV Male—164 Female—108 Age—63 ± 11 years Non-NIV Male—79 Female—101 Age—66 ± 11 years	At each clinic visit, subjects reported average hours of daily NIV use since last visit.	Type of onset

**Table 2:** Risk of bias of included studies.

Author (Year)	Random Sequence Generation (Selection Bias)	Allocation Concealment (Selection Bias)	Groups Similar to Baseline	Outcome Measurements Similar to Baseline	Outcome Measurements (Detection Bias)	Incomplete Outcome Data (Attrition Bias)	Selection Outcome Reporting (Reporting Bias)	Protection Against Contamination	Other Bias
Bourke SC et al., 2006 [13]	Low	Low	Unclear	N/A	Low	Low	Low	Unclear	High
Waltteri Siirala et al., 2013 [21]	High	High	Low	N/A	Low	Low	Low	High	Low
Berlowitz DJ et al., 2015 [22]	High	High	High	N/A	Low	Low	Low	Unclear	High
Khamankar N. et al., 2018 [23]	High	High	Unclear	N/A	Low	Low	Low	Unclear	High
Jesus Sancho et al., 2018 [24]	High	High	High	N/A	Low	Low	Low	Unclear	Low
Walsh LJ et al., 2021 [25]	High	High	High	N/A	Low	Low	Low	Unclear	High
Ackrivo J et al., 2021 [26]	High	High	Low	N/A	Low	High	Low	Unclear	High

N/A—Not Applicable.

**Table 3:** Survival of ALS patients on NIV and non-NIV treatment.

Study	Survival (Years, Months, Days)		
	NIV	Non-NIV	*p*
Bourke SC et al., 2006 [13]	219 days (75–1382)	171 days (1–878)	0.0062
Waltteri Siirala et al., 2013 [21]	22 months (3–65)	8 months (1–26)	<0.001
Berlowitz DJ et al., 2015 [22]	28.63 months	15.02 months	0.001
Khamankar N. et al., 2018 [23]	21.03 months (IQR = 23.97 months)	13.84 months (IQR = 11.97 months)	<0.001
Jesus Sancho et al., 2018 [24]	43 months (38.13–47.86)	28 months (21.49–34.57)	0.001
Walsh LJ et al., 2021 [25]	28 months	12 months	0.002
Ackrivo J et al., 2021 [26]	7.4 (IQR, 2.7–15.5) months	8.0 (IQR, 3.5–15.5) months	0.67

IQR—interquartile range.

## Data Availability

All data are presented in the form of this article.
